# A retrospective analysis of serous effusions based on the newly proposed international system for reporting serous fluid cytopathology: a report of 3633 cases in an oncological center

**DOI:** 10.1186/s13000-022-01241-4

**Published:** 2022-07-02

**Authors:** Yan-li Zhu, Wen-hao Ren, Qian Wang, Hai-zhu Jin, Yi-yi Guo, Dong-mei Lin

**Affiliations:** Key Laboratory of Carcinogenesis and Translational Research (Ministry of Education), Department of Pathology, Peking University Cancer Hospital and Institute, Beijing, 100142 China

**Keywords:** Cytology, Effusion, The International System for Reporting Serous Fluid, Risk of malignancy, Liquid-based cytology

## Abstract

**Background:**

The International System for Reporting Serous Fluid Cytopathology (TIS) was recently proposed. We retrospectively applied TIS recommendations for reporting the cytological diagnosis of serous effusions and reported our experience.

**Methods:**

All the serous effusions from January 2018 to September 2021 were retrieved from the database. Recategorization was performed using the TIS classification, the risk of malignancy (ROM) was calculated for each TIS category. In addition, on the basis of the original TIS classification, we further subdivided the TIS category IV (suspicious for malignancy, SFM) into 2 groups (IVa and IVb) according to cytological characteristics (quality and quantity) to explore the necessity of SFM subclassification. The performance evaluation was carried out using different samples (pleural, peritoneal and pericardial effusions) and preparation methods (conventional smears, liquid-based preparations and cell blocks).

**Results:**

A total of 3633 cases were studied: 17 (0.5%) were diagnosed as ‘nondiagnostic’ (I, ND), 1100 (30.3%) as ‘negative for malignancy’ (II, NFM), 101 (2.8%) as ‘atypia of undetermined significance’ (III, AUS), 677 (18.6%) as ‘suspicious for malignancy’ (IV, SFM), and 1738 (47.8%) as ‘malignant’ (V, MAL). The ROMs for the categories were 38.5%, 28.6%, 52.1%, 99.4% and 100%, respectively. The ROM for SFM was significantly higher than that for AUS (*P* < 0.001), while the difference between the ROMs for IVa and IVb was insignificant. The sensitivity, negative predictive value (NPV) and diagnostic accuracy of liquid-based preparations were all superior to those of conventional smears and cell blocks in detecting abnormalities. Using the three preparation methods simultaneously had the highest sensitivity, NPV and diagnostic accuracy.

**Conclusion:**

Serous effusion cytology has a high specificity and positive predictive value (PPV), and TIS is a user-friendly reporting system. Liquid-based preparations could improve the sensitivity of diagnosis, and it is best to use three different preparation methods simultaneously for serous effusion cytologic examination.

## Background

Effusions can be produced inside serous cavities in neoplastic and nonneoplastic lesions. Serous effusion cytology is a common clinical examination method to distinguish benign and malignant serous effusions due to its advantages of being minimally invasive, easily available, and cost-effective [1–3]. In view of the importance of cytology in the evaluation of effusion specimens, its role in patient management has become increasingly important. An international group of cytopathology experts published “The International System for Reporting Serous Fluid Cytopathology” (TIS) to standardize the reporting terminology and criteria to establish diagnostic categories with high diagnostic value [4, 5].

The 5 proposed diagnostic categories are nondiagnostic (I, ND), negative for malignancy (II, NFM), atypia of undetermined significance (III, AUS), suspicious for malignancy (IV, SFM), and malignant (V, MAL). TIS has defined the AUS category as specimens that lack quantitative or qualitative cytologic features to be confidently diagnosed as either benign or malignant and that exhibit sufficiently clear morphologic features to exclude the possibility of classifying them as ND. The SFM category is defined as specimens showing cytologic features usually found in malignant lesions but insufficient either in quality or quantity for a definitive diagnosis of malignancy. Hou et al. [6] showed that the risk of malignancy (ROM) for SFM was significantly higher than that for AUS (*P* < 0.01), which supports the separate diagnostic categories of these two independent groups. A key question to ask is whether SFM category based on heterogeneous cytological features (quality or quantity) carries the same ROM and deserves the same clinical management.

To date, only a few publications have supported the use of a particular terminology for serous effusion cytology [6–15]. In this study, we retrospectively applied TIS recommendations for reporting the cytological diagnosis of serous effusions. After sample reclassification, the ROM for each TIS category was calculated, and the performance evaluation was carried out between different sample preparations (conventional smears, liquid-based preparations and cell blocks). To the best of our knowledge, this is the first publication looking into the method of preparation employed in a retrospective cohort of serous effusions based on the TIS. In addition, we sought to review and subclassify the SFM category into 2 groups (IVa and IVb) by the cytological features (quality and quantity) to calculate the ROM of each subgroup and to evaluate the necessity of having a subclassification for the SFM category.

## Material and methods

### Data collection

The study was approved by the Ethics Committee of the Peking University Cancer Hospital. The inclusion criteria were cytopathological samples of serous effusions (pleural, peritoneal and pericardial effusions) from Peking University Cancer Hospital from January 2018 to September 2021. Due to the artificial/iatrogenic origin, peritoneal washings were excluded from the current study. Data were collected from pathology databases and electronic medical records, including patient demographics, clinical presentation, cytology and histology reports, ancillary studies and patient management. The cases were reclassified based on the microscopic description of the sample, the final diagnosis and codification of the cytology report. If the information contained in the report was considered insufficient, the original slides were reviewed by two experienced cytopathologists (Yanli Zhu and Wenhao Ren) and classified in the most suitable TIS category. All cases were reclassified blindly by the two cytopathologists according to the criteria defined in the TIS. When two cytopathologists did not agree on the reclassification of a particular case, they reached a consensus after discussion.

### Preparation of the specimens

Specimens were received fresh and were either entirely submitted for centrifugation or a representative 100 ml sample was processed. During processing, the samples were divided into 2 tubes and centrifuged at 2500 revolutions per minute for 10 min. In addition, the supernatant was decanted. One of the tubes was prepared as conventional smears stained with hematoxylin–eosin and as liquid-based cytology samples using the ThinPrep method stained with Papanicolaou stain. A cell pellet was obtained from the other tube and the material was fixed in formalin, processed as a cell block, and stained with hematoxylin–eosin. A cell block is routinely prepared for all samples unless there is inadequate material.

### Criteria used for each TIS category

Recategorization was performed using the TIS classification, and cases were allocated to one of the five proposed categories. The following criteria were used for reclassification:

- I. Non-diagnostic (ND): Specimens with insufficient cellular elements for cytologic interpretation. Generally, it would be reasonable to consider a specimen ND due to scant cellularity or excess degeneration, improper preservation and obscuring blood in the serous effusion specimens.
- II. Negative for malignancy (NFM): Specimens with cellular changes that completely lack evidence of mesothelial or non-mesothelial malignancy. The morphology of the cells, including mesothelial cells, macrophages, lymphocytes, and polymorphs, were benign irrespective of the clinical history and imaging studies.
- III. Atypia of undetermined significance (AUS): Specimens that lack quantitative or qualitative cytologic features to be confidently diagnosed as either benign or malignant and that exhibit sufficiently clear morphologic features to exclude the possibility of classifying them as ND. The atypical morphologic features expressed will more closely approximate benign, reactive, or degenerative features than malignant features.
- IV. Suspicious for malignancy (SFM): Specimens showing cytologic features usually found in malignant lesions but insufficient either in quality or quantity for a definitive diagnosis of malignancy. The diagnosis of IVa was made when there were rare cells displaying moderate-to-severely atypical features that were qualitatively insufficient to confidently exclude malignancy. And the diagnosis of IVb was made when there were rare cells displaying severely atypical features that were suspicious for malignancy but quantitatively insufficient for ancillary studies.
- V. Malignancy (MAL): Specimens showing cytomorphologic features that, either alone or combined with the results from ancillary studies, are diagnostic of a primary (mesothelioma) or secondary (metastatic) malignancy. This category, irrespective of the history, shows medium to high cellularity with malignant cells in clusters and scattered singly on the cytosmears.

### Statistical analysis

Statistical analysis was performed using IBM SPSS Statistics (Version 20.0; IBM Corp., New York, USA). The variables were mainly categorical, and the test used was the chi-square test. A *P* value less than 0.05 was considered significant. The gold standard for true diagnosis was based on a histological diagnoses or clinical diagnoses. The histological diagnoses were the biopsy or postoperative pathological results of the pleura, peritoneum, or pericardium corresponding to the effusion, and the clinical diagnoses were made in combination with clinical manifestations, laboratory results and medical imaging examination results. All histological and clinical diagnoses were performed independently and blindly by two physicians. For cases with inconsistent results, the same diagnosis was made after discussion by two physicians.

Performance analysis included the calculation of sensitivity, specificity, positive predictive value (PPV), negative predictive value (NPV) and diagnostic accuracy for different samples (pleural, peritoneal and pericardial effusion cytology samples) and preparation methods (conventional smears, liquid-based preparations and cell blocks). During performance analysis, the results were calculated separately according to MAL as positive, MAL and SFM as positive, MAL, SFM and AUS as positive. ND cytology samples were excluded from performance analysis; In some cases, necessary laboratory tests and medical imaging examinations were performed, but if it was still unclear whether the serosa was invaded, and those cases were also excluded from performance analysis sequence.

## Results

### Patient demography and clinicopathologic data

Between January 2018 and September 2021, a total of 3633 serous effusions were diagnosed at our institution, including 2366 (65.1%) pleural effusions, 1150 (31.7%) peritoneal effusions and 117 (3.2%) pericardial effusions. The mean age and male to female ratio were 58.7 (range 9–93 years) and 0.96, respectively. The volume of serous effusions ranged from 20 to 1000 ml (mean: 265 ml). Cell blocks were prepared in 2882 cases. Of all specimens, 17 (0.5%) were diagnosed as ND, 1100 (30.3%) as NFM, 101 (2.8%) as AUS, 677 (18.6%) as SFM, and 1738 (47.8%) as MAL. The patient demographics and specimen characteristics are shown in Table 1.

**Table 1:** Patient demographics and specimen characteristics of 3633 serous effusions based on each TIS category

Diagnostic category	ND	NFM	AUS	SFM	MAL	Total
Number of patients(n, percentage)	17(0.5%)	1100(30.3%)	101(2.8%)	677(18.6%)	1738(47.8%)	3633
Outpatient/inpatient	5/12	203/897	24/77	158/519	436/1302	826/2807
Gender(number of men/women)	11/6	656/444	66/35	341/336	703/1035	1777/1856
Average age(year, ranges)	53(40–65)	59(17–93)	61(9–90)	59(17–93)	59(14–92)	58.7(9–93)
Cell block slides(n)	12	637	72	518	1643	2882
Serous effusion source(n)						
*Pleural*	14	723	77	442	1110	2366
*Peritoneal*	2	331	24	222	571	1150
*Pericardial*	1	46	0	13	57	117
Volume: median(range)	200(50–600)	262(20–1000)	260(30–1000)	256(25–1000)	273(25–1000)	265(20–1000)

Abbreviations: *ND* non-diagnostic, *NFM* negative for malignancy, *AUS* atypia of undetermined significance, *SFM* suspicious of malignancy, *MAL* malignancy

### Risk of malignancy

Forty-five cases were excluded from the ROM analysis because the corresponding gold standard failed to give a clear diagnosis of benign and malignant lesions. Ultimately, a total of 3588 cases were used to calculate ROM for each category. Table 2 depicts the calculated ROM for each category, and Table 3 provides the subclassification of the indeterminate categories of AUS and SFM and the corresponding ROMs.

**Table 2:** The risk of malignancy in current study and comparison with a few previous publications

Author	Specimen type	Year	Total cases	Risk of malignancy(ROM)				
				ND	NFM	AUS	SFM	MAL
*Sahar J. F et al* [15]	PF + AF + PeriF	2019	34,941	17.4%	20.7%	65.9%	81.8%	98.9%
*Ediel V et al* [14]	PF	2019	519	50%	44%	50%	83.3%	96.2%
*Cláudia L et al* [10]	PF/AF/ PeriF	2020	1496/763/64	57.1%/100%/-	23.9%/26.3%/0%	50%/62.5%/0%	76.2%/91.7%/-	100%/100%/100%
*Yi X et al* [11]	PF	2021	2454	26.7%	12%,	62.3%	77.8%	100%
*Shilpy J et al* [12]	PF	2021	939	87.5%	51.6%	88.2%	87.5%	100%
*Daniel P et al* [13]	PF	2021	350	40%	20.16%	42.86%	78.57%	100%
*Tieying H et al* [6]	PF + AF + PeriF	2021	2405	-	-	39%	64%	-
Current study	PF + AF + PeriF	2021	3588	38.5%(5/13)	28.6%(304/1064)	52.1%(50/96)	99.4%(673/677)	100%(1738/1738)
	PF		2326	40%(4/10)	29.8%(206/691)	49.3%(36/73)	99.3%(439/442)	100%(1110/1110)
	AF		1145	0%(0/2)	27.5%(90/327)	60.9%(14/23)	99.5%(221/222)	100%(571/571)
	PeriF		117	100%(1/1)	17.4%(8/46)	0%(0/0)	100%(13/13)	100%(57/57)

Abbreviations: *ND* non-diagnostic, *NFM* negative for malignancy, *AUS* atypia of undetermined significance, *SFM* suspicious of malignancy, *MAL* malignancy, *PF* pleural fluid, *AF* ascitic fluid, *PeriF* pericardial fluid

**Table 3:** Subclassification of the indeterminate categories of AUS and SFM and the corresponding risk of malignancy(ROM)

Subclassification of AUS/SFM	No.(%)	Surgical pathology/clinical diagnosis		ROM
		**Benign**	**Malignant**	
AUS	96	46	50	52.1%
SFM	677	4	673	99.4%
*SFM-A*	333	4	329	98.8%
*SFM-B*	344	0	344	100%
Total	773	50	723	93.5%

SFM-A: There were rare cells displaying moderate-to-severely atypical features that were qualitatively insufficient to confidently exclude malignancy; SFM-B: There were rare cells displaying severely atypical features that were suspicious for malignancy but quantitatively insufficient for ancillary studies

Abbreviations: *AUS* atypia of undetermined significance, *SFM* suspicious of malignancy

### The ROM and performance analysis in pleural, peritoneal and pericardial effusion specimens

#### Pleural effusions

**Fig. 1:**
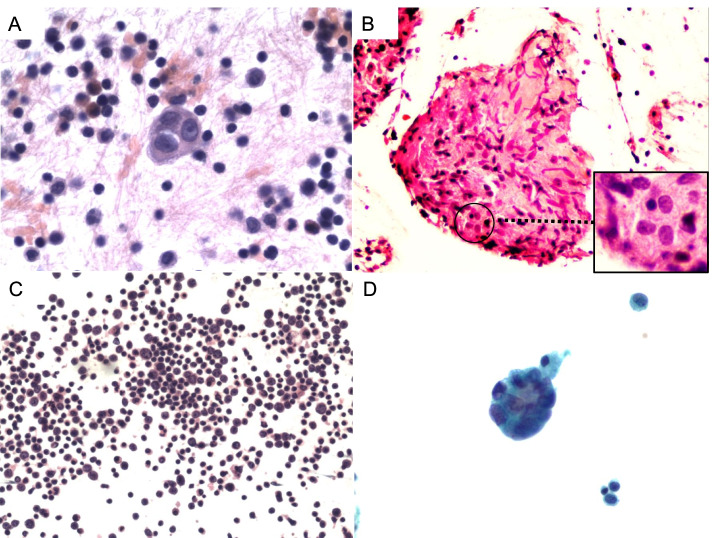
False-positive cases in pleural effusion specimens. Case 1: The cytological diagnosis was ‘SFM (IVa)’ (**A**, HE, × 400), while the histological diagnosis was granulomatous lesions (**B**, HE, × 200). We think that the epithelioid cells in the granulomatous lesions (bottom right of the Figure B) may have been mistaken for SFM. Case 2: The cytological diagnosis was ‘SFM (IVa)’ (**C**, HE, × 100), while the clinical diagnosis was ‘tuberculosis’. The patient had a history of abdominal lymphoma. A few weeks after the cytological diagnosis, he was clinically diagnosed with thoracic tuberculosis, and the pleural fluid disappeared after anti-tuberculosis treatment. The history of lymphoma may have led to our incorrect diagnosis. Case 3: The cytological diagnosis was ‘SFM (IVa)’ (**D**, Papanicolaou stain, × 400), while the clinical diagnosis was ‘chylothorax’. The misdiagnosed cells may be reactive mesothelial cells caused by chylothorax

In total, 2326 cases of pleural effusions were reclassified in accordance with criteria set by the TIS: 10 (0.4%) ND, 691 (29.7%) NFM, 73 (3.1%) AUS, 442 (19.0%) SFM, and 1110 (47.7%) MAL. When considering only MAL as positive, false negatives were found in 681 cases, while there were no false-positive cases. When considering MAL and SFM as positive, false negatives were found in 242 cases, while three false-positive cases were found (Fig. 1). Possible reasons for those false-positives are outlined in Fig. 1. The ROM was 40% (4/10) for ND, 29.8% (206/691) for NFM, 49.3% (36/73) for AUS, 99.3% (439/442) for SFM and 100% (1110/1110) for MAL. Considering only MAL as positive cases, the sensitivity was 62.0%, specificity was 100%, PPV was 100%, NPV was 43.5% and diagnostic accuracy was 70.6%. Considering MAL and SFM as positive, the sensitivity was 86.5%, specificity was 99.4%, PPV was 99.8%, NPV was 68.3% and diagnostic accuracy was 89.4%. Considering MAL, SFM and AUS as positive, the sensitivity was 88.5%, specificity was 92.4%, PPV was 97.5%, NPV was 70.2% and diagnostic accuracy was 89.4%.

#### Peritoneal effusions

**Fig. 2:**
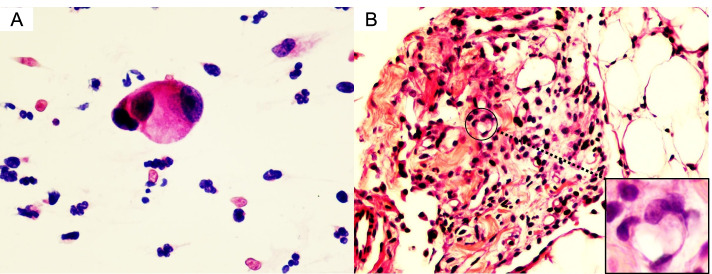
The false-positive case in peritoneal effusion specimens. The cytological diagnosis was ‘SFM (IVa)’ (**A**, HE, × 400), while the omentum biopsy result was chronic inflammation (**B**, HE, × 200). We think that the fibroblasts (bottom right of Figure B) may have been mistaken for SFM

In total, 1145 peritoneal effusion samples were reclassified by the TIS: 2 (0.2%) ND, 327 (28.6%) NFM, 23 (2.0%) AUS, 222 (19.4%) SFM and 571 (49.9%) MAL. Considering only MAL as positive, false negatives were found in 325 cases, while there were no false-positive cases. When considering MAL and SFM as positive, false negatives were found in 104 cases, while one false-positive case was found (Fig. 2), A possible reason for the false-positive was outlined in the description in Fig. 2. The ROM was 0% (0/2) for ND, 27.5% (90/327) for NFM, 60.9% (14/23) for AUS, 99.5% (221/222) for SFM and 100% (571/571) for MAL. Considering only MAL as positive cases, the sensitivity was 63.7%, specificity was 100%, PPV was 100%, NPV was 43.2% and diagnostic accuracy was 71.6%. Considering MAL and SFM as positive, the sensitivity was 88.4%, specificity was 99.6%, PPV was 99.9%, NPV was 70.3% and diagnostic accuracy was 90.8%. Considering MAL, SFM and AUS as positive, the sensitivity was 90.0%, specificity was 96.0%, PPV was 98.8%, NPV was 72.5% and diagnostic accuracy was 91.3%.

#### Pericardial effusions

A total of 117 cases were reclassified by the TIS: 1 (0.9%) ND, 46 (39.3%) NFM, 13 (11.1%) SFM and 57 (48.7%) MAL, and there were no cases of AUS. When considering MAL as positive and MAL and SFM as positive, there were both no false-positive cases, and false negatives were found in 21 cases and 8 cases, respectively. The ROM was 100% (1/1) for ND, 17.4% (8/46) for NFM, 100% (13/13) for SFM and 100% (57/57) for MAL. Considering only MAL as positive cases, the sensitivity was 73.1%, specificity was 100%, PPV was 100%, NPV was 64.4% and diagnostic accuracy was 81.9%. Considering MAL and SFM as positive, the sensitivity was 89.7%, specificity was 100%, PPV was 100%, NPV was 82.6% and diagnostic accuracy was 93.1%. Considering MAL, SFM and AUS as positive, the sensitivity was 89.7%, specificity was 100%, PPV was 100%, NPV was 82.6% and diagnostic accuracy was 93.1%. Table 4 presents the results of the performance analysis among different serous effusions in the current study and previous publications.

**Table 4:** The performance analysis among different serous effusions in current study and previous publications

			Positive standard		
			MAL + SFM + AUS	MAL + SFM	MAL
Pleural effusions	Current study	Sensitivity	88.5%	86.5%	62.0%
		Specificity	92.4%	99.4%	100.0%
		PPV	97.5%	99.8%	100.0%
		NPV	70.2%	68.3%	43.5%
		Diagnostic accuracy	89.4%	89.4%	70.6%
	*Previous publications*	*Sensitivity*	*-*	*60.3%-99.7% *[11, 13]	*61.6%-87% *[10, 11, 14, 16]
		*Specificity*	*-*	*98.6%-99.4% *[11, 13]	*93.3%-100% *[10, 11, 14, 16]
		*PPV*	*-*	*96.5%-98.3% *[11, 13]	*96.2%-100% *[10, 11, 14, 16]
		*NPV*	*-*	*79.2%-99.9% *[11, 13]	*56%-98% *[10, 11, 14, 16]
		*Diagnostic accuracy*	*-*	*97% *[11]	*81.3%-98% *[10, 11, 14, 16]
Peritoneal effusions	Current study	Sensitivity	90.0%	88.4%	63.7%
		Specificity	96.0%	99.6%	100.0%
		PPV	98.8%	99.9%	100.0%
		NPV	72.5%	70.3%	43.2%
		Diagnostic accuracy	91.3%	90.8%	71.6%
	*Previous publications*	*Sensitivity*	*-*	*-*	*61.2% *[10]
		*Specificity*	*-*	*-*	*100.0% *[10]
		*PPV*	*-*	*-*	*100.0% *[10]
		*NPV*	*-*	*-*	*70.0% *[10]
		*Diagnostic accuracy*	*-*	*-*	*79.7% *[10]
Pericardial effusions	Current study	Sensitivity	89.7%	89.7%	73.1%
		Specificity	100.0%	100.0%	100.0%
		PPV	100.0%	100.0%	100.0%
		NPV	82.6%	82.6%	64.4%
		Diagnostic accuracy	93.1%	93.1%	81.9%
	*Previous publications*	*Sensitivity*	*-*	*-*	*97%-100% *[10, 16]
		*Specificity*	*-*	*-*	*100% *[10, 19]
		*PPV*	*-*	*-*	*100% *[10, 19]
		*NPV*	*-*	*-*	*99%-100% *[10, 16]
		*Diagnostic accuracy*	*-*	*-*	*99%-100% *[10, 16]
Total effusions	Current study	Sensitivity	89.0%	87.2%	62.9%
		Specificity	93.8%	99.5%	100.0%
		PPV	98.0%	99.8%	100.0%
		NPV	71.4%	69.5%	44.1%
		Diagnostic accuracy	90.1%	90.0%	71.3%
	*Previous publications*	*Sensitivity*	*23.5%-100% *[15]		
		*Specificity*	*66.2%-100% *[15]		
		*PPV*	*87.0%-100% *[15]		
		*NPV*	*19.1%-100% *[15]		
		*Diagnostic accuracy*		*-*	

Abbreviations: *AUS* atypia of undetermined significance, *SFM* suspicious for malignancy, *MAL* malignancy, *PPV* positive predictive value, *NPV* negative predictive value

#### Performance analysis among different sample preparations

**Fig. 3:**
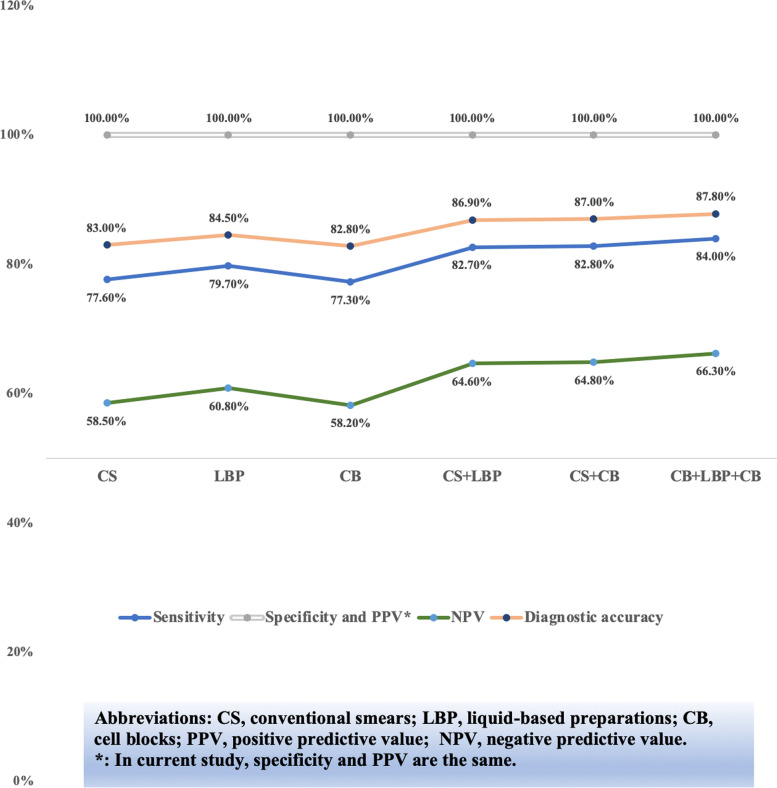
The performance evaluation among different sample preparations in 1288 cases when considering malignancy, suspicious of malignancy and atypia of undetermined significance as positive

**Fig. 4:**
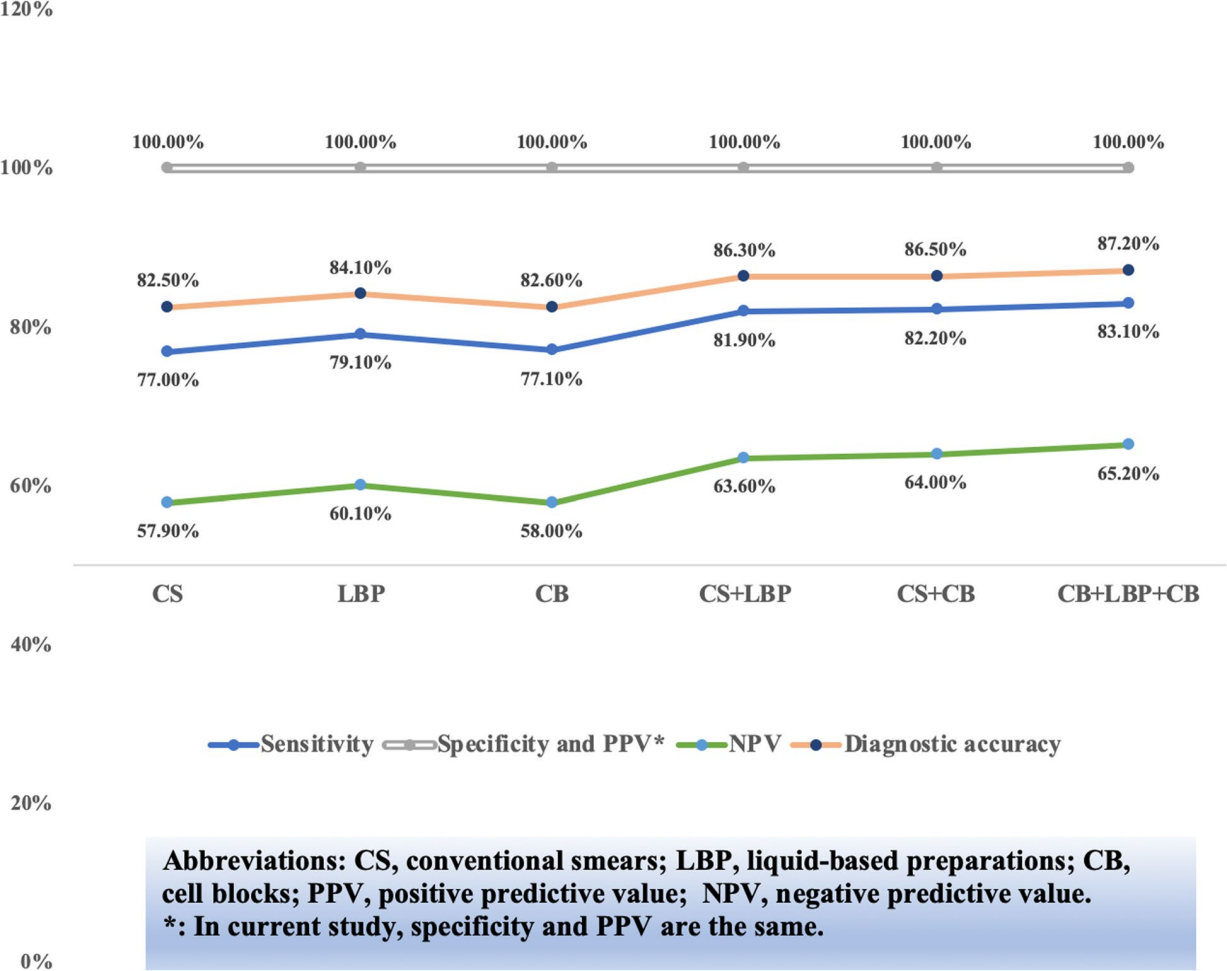
The the performance evaluation among different sample preparations in 1288 cases when considering malignancy and suspicious of malignancy as positive

A total of 1288 cases with both conventional smears, liquid-based preparations and cell blocks diagnoses were collected and the performance evaluation among different preparation methods was analyzed. In our analysis, the sensitivity, NPV and diagnostic accuracy of liquid-based preparations were all superior to conventional smears and cell blocks in detecting abnormalities (Fig. 3 and Fig. 4). Besides, we found that applying two methods at the same time preceded to any single method and using three methods at the same time had the highest sensitivity, NPV and diagnostic accuracy, while there was little difference between the two diverse methods of preparation (Fig. 3 and Fig. 4).

## Discussion

Serous effusion cytology is a minimally invasive and cost-effective diagnostic method used to investigate the etiologies of body cavity effusions and can guide clinical decision-making. To the best of our knowledge, this is the largest series to date of such analyses in serous effusions. We evaluated our application of the recently proposed TIS on reporting serous effusion cytopathology. A total of 3633 patients were included, among which 17 (0.5%), 1100 (30.3%), 101 (2.8%), 677 (18.6%) and 1738 (47.8%) cases were classified into ND, NFM, AUS, SFM and MAL groups, respectively.

The malignancy rate (47.8%) detected in our cohort was higher than that reported in the literature, which ranges between 4% and 22.4% [6–9, 11–13, 15], but was similar to other reports in the literature from oncological centers [6, 10, 14]. The percentage of our SFM cases was also slightly higher than that in other reported studies (range: SFM, 1.3%-6.3%) [6–15]. These results are characteristic of an oncological center, where neoplastic conditions are the main cause of cytological examination of body fluids. If a case is diagnosed as SFM, combined with the tumor history and clinical symptoms, the clinical doctor will diagnose the case as positive and proceed directly to the next step of treatment, which makes some SFM lose the opportunity to be identified as MAL by immunocytochemistry and other auxiliary methods. In addition, our study included both outpatient and hospitalized patients, and the outpatients often failed to be identified as malignant by auxiliary examination. Moreover, we did not use a specific number of cells to determine whether a sample was suspicious or positive for malignancy. In patients with a clear history of disseminated malignancy, although there was no immunochemistry support, a few highly atypical cells might be sufficient to diagnose SFM, while in other clinical situations, the same number of cells might not be enough for a diagnosis of SFM and can only be diagnosed as AUS. If we follow the principles of clinical management, 96.6% of our serous effusions got a therapeutically meaningful diagnosis, including NFM, MAL and SFM, and only a small proportion of cases were diagnosed as ND and AUS (0.5%, 2.7%).

Our nondiagnostic rate was 0.5%. This is in line with other reports in the literature, which present nondiagnostic rates of 0% to 5.6% in serous effusion [6–15]. The cases classified as ND in this series were all due to scant cellularity or excess degeneration, improper preservation and obscuring blood in the serous effusion specimens. Therefore, whole blood samples should be anticoagulated in a timely manner before sample preparation.

The minimal threshold of adequacy for fluid interpretation is still contentious and has not been described clearly in the TIS. Some earlier studies have suggested a minimum of 50–75 ml [17–19], but the evidence is limited. Recently, Gokozan et al. [8] performed a root cause analysis of the diagnoses of atypia or suspicious for malignancy and showed that 50 mL and below were considered low volume samples, and were included as a root cause for indeterminate diagnoses. In the present study, the 17 nondiagnostic cases all had a specimen volume greater than or equal to 50 ml and many of our malignant cases had a very low volume submitted to our laboratory. Therefore, the volume threshold of adequacy should be regarded as a recommendation, with the final decision left to each practice.

In our cohort, the ROM values of serous effusions in the ND, NFM, AUS, SFM and MAL groups were 38.5%, 28.6%, 52.1%, 99.4% and 100%, respectively. It is worth noting that high ROMs can be seen in our study for SFM. One possible reason for this result could be due to the fact that our data come from a large oncological center, and the majority of the serous effusions often originate from tumors, providing a potential selection bias. Our high ROM for SFM supports the viewpoint that most clinicians will manage patients with SFM effusions similar to those with a malignant diagnosis. Besides, in our study, the ROMs for the nondiagnostic and negative categories were also high, which may also be attributed to the nature of the patient population in our cancer center, many of whom were referred to our hospital with an established malignant diagnosis and frequently at an advanced stage of disease. For the cases with high clinical suspicion, even though cytology was negative, patients usually underwent medical imaging examination and clinicians tended to pursue pleural biopsies; therefore, the ROMs for the nondiagnostic and negative categories will improve.

The TIS describes two indeterminate categories, AUS and SFM, created to encompass all of the fluid that could not be placed under the NFM or MAL categories. In our study, the ROM for SFM was significantly higher than that for AUS (*P* < 0.001), thus providing support for retaining the two indeterminate categories as independent ones. The difference between the ROMs for the IVa and IVb was insignificant with a *P* value of 0.124, which indicates that although different situations exist that can be diagnosed in the SFM category, there is no need to reclassify for the SFM category. From the Table 2, it is worth noting the wide range of ROM calculated for each diagnostic category, which is likely attributable to the variation in reporting among different institutions. Standardized reporting would also provide a meaningful language that clinicians can uniformly understand and utilize in their patient management. More research is needed to convey the ROM of each category to the corresponding clinical colleagues to optimize patient care.

Our performance analysis was in agreement with previous publications [10, 11, 13, 14, 16] (Table 4). By comparing the performance evaluation of different groups (considering cytological diagnosis of MAL + SFM + AUS as positive, considering cytological diagnosis of MAL + SFM as positive, and considering cytological diagnosis of MAL as positive), it is best to consider the categories of MAL and SFM as positive, while excluding AUS, to be beneficial to clinical management. This is supported by our data (Table 4) and agrees with the perspective of TIS [5]. Moreover, it is worth mentioning that when considering the categories of MAL and SFM as positive, there were only 4 false-positive cases, and the 4 cases were all diagnosed as IVa, and the total specificity and the PPV were as high as 99.5% and 99.8%, respectively.

Compared with conventional smears, liquid-based preparations require less skill, and more importantly, allow for the application of ancillary examinations [20]. Moreover, liquid-based preparations permit fa more even distribution of cells over the slide area, a reduction in obscuring background elements, and better preservation of nuclear detail and cytoplasm. Several studies have shown that slides prepared with liquid-based preparations have a lower nondiagnostic incidence and higher accuracy than conventional smears [21–23]. In our study, the sensitivity, NPV and diagnostic accuracy of liquid-based preparations were all superior to conventional smears and cell blocks in detecting abnormalities. In addition, by comparing the performance evaluation among different methods of preparation, we found that applying two methods at the same time was superior to any single method and using three methods simultaneously had the highest sensitivity, NPV and diagnostic accuracy, while there was little difference between the two diverse methods of preparation. Therefore, in terms of the selection of preparation methods, it is best to use three different methods simultaneously for serous effusion cytologic examination.

There are 2 main limitations in our study. The first is attributed to the nature of the patient population in our cancer center, leading to a high rate of MAL and SFM, as well as a high ROM for each category. Another limitation of our study is that it was a single-center and retrospective study, and cytological diagnoses were interpreted by only two cytopathologists, who, although they reclassified cases according to TIS criteria, may still have individual biases.

## Conclusions

In conclusion, this study supports the idea of retaining the two indeterminate categories (AUS and SFM) as independent ones, and there is no need to reclassify for the SFM category. By comparing the performance evaluation of different groups, it is best to consider the categories of MAL and SFM as positive, while excluding AUS. A total of 96.6% of our serous effusions received a directed diagnosis, including NFM, MAL and SFM, and only a small proportion of cases were diagnosed as ND and AUS (0.5%, 2.7%), demonstrating that TIS is a user-friendly reporting system. The sensitivity, NPV and diagnostic accuracy of liquid-based preparations were all superior to those of conventional smears and cell blocks in detecting abnormalities. It is best to use three different preparation methods simultaneously for serous effusion cytologic examination.

## Data Availability

The datasets used and/or analyzed during the current study are available from the corresponding author on reasonable request.

## Abbreviations

AF: Ascitic fluid
AUS: Atypia of undetermined significance
MAL: Malignancy
ND: Non-diagnostic
NFM: Negative for malignancy
NPV: Negative predictive value
PeriF: Pericardial fluid
PF: Pleural fluid
PPV: Positive predictive value
ROM: Risk of malignancy
SFM: Suspicious of malignancy
TIS: The International System for Reporting Serous Fluid Cytopathology
